# Development and evaluation of a bladder Cancer specific survivorship care plan by patients and clinical care providers: a multi-methods approach

**DOI:** 10.1186/s12913-020-05533-7

**Published:** 2020-07-24

**Authors:** Cheryl T. Lee, Nihal E. Mohamed, Sailaja Pisipati, Qainat N. Shah, Piyush K. Agarwal, Tracy M. Downs, Michael Droller, Scott M. Gilbert, Heather H. Goltz, Simon J. Hall, Mohamed Hendawi, Jean Hoffman-Censits, Michael O’Donnell, Matthew Kaag, Lawrence I. Karsh, Wassim Kassouf, Diane Z. Quale, Arthur Sagalowsky, Gary D. Steinberg, David M. Latini

**Affiliations:** 1 Department of Urology, The Ohio State University, Columbus, OH USA; 2 Department of Urology and Oncological Sciences, Icahn School of Medicine at Mount Sinai, 1 Gustave L Levy Place, New York, NY 10029 USA; 3 University of Nevada Reno School of Medicine, Reno, NV USA; 4 National Cancer Institute, Bethesda, MD USA; 5 University of Wisconsin, Madison, WI USA; 6 Moffitt Cancer Center, Tampa, FL USA; 7 Social Work Program, University of Houston-Downtown, Houston, TX USA; 8 Section of Infectious Diseases, Baylor College of Medicine, Houston, TX USA; 9 Smith Institute for Urology, Hofstra School of Medicine/Northwell Health System, Lake Success, NY USA; 10 Sidney Kimmel Cancer Center at John Hopkins, Baltimore, MD USA; 11 University of Iowa, Iowa City, IA USA; 12 Penn State Health, Hershey, PA USA; 13 The Urology Center of Colorado, Denver, CO USA; 14 McGill University Health Center, Montreal, Canada; 15 Bladder Cancer Advocacy Network, Bethesda, MD USA; 16 University of Texas, Southwestern, Dallas, TX USA; 17 NYU Langone Health, New York, NY USA; 18 Baylor College of Medicine, Houston, TX USA

**Keywords:** Survivorship care plan, Bladder Cancer specific survivorship care plan, Focus groups, Care providers, Muscle invasive bladder Cancer, Non-muscle invasive bladder Cancer

## Abstract

**Background, context and purpose:**

In spite of the mixed evidence for their impact, survivorship Care Plans (SCPs) are recommended to enhance quality of care for cancer survivors. Data on the feasibility of SCPs in bladder cancer (BC) is sparse. Using a mixed-methods approach, this study describes the iterative development, acceptability and feasibility of BC specific SCP (BC-SCP) in clinical settings.

**Methods:**

In Phase I, we developed the BC-SCP. In Phase II, we conducted four focus groups with 19 patients and 15 providers to examine its acceptability and usability challenges. Data analyses using the Atlas.ti program, informed refinement of the BC-SCP. In Phase III, we conducted feasibility testing of the refined BC-SCP with 18 providers from 12 health-centers. An encounter survey was completed after each assessment to examine the feasibility of the BC-SCP. Chi-square and Fisher Exact tests were used for comparative analyses.

**Results:**

During phase I, we observed high patient and provider acceptability of the BC-SCP and substantial engagement in improving its content, design, and structure. In Phase II, providers completed 59 BC-SCPs. Mean time for BC-SCP completion was 12.3 min. Providers reported that BC-SCP content was clear, did not hamper clinic flow and was readily completed with easy-to-access information. Comparative analyses to examine differences in SCP completion time by patient clinico-demographic characteristics and provider type revealed no significant differences.

**Conclusions:**

Our BC-SCP has clinical relevance, and can be used in an active practice setting. However, considerable progress will be necessary to achieve implementation of and sharing the BC-SCP with patients and care providers, particularly within the electronic medical record. In summary, BC-SCPs are essential to improve the follow up care of BC survivors. Clinical resources are required to ensure appropriate implementation of BC-SCPs.

**Trial registration:**

Study HUM00056082.

## Background

Bladder cancer (BC) is the second most common genitourinary cancer in the United States accounting for 4.5% of all new cancer cases worldwide [1]. The diagnosis and management of BC causes significant psychological stress due to invasive treatments, short- and long-term side effects, life-long self-care requirements (e.g., utility of stomal appliances and catheterization), and extensive cancer surveillance [2]. We and others have shown that significant unmet informational needs and supportive care needs persist throughout the BC trajectory including survivorship [2–4]. Although the need for information about BC, treatment options, short-term side-effects, and self-care skills is readily apparent after cancer diagnosis and treatment, the need for management of long-term side-effects (e.g., urinary incontinence, sexual dysfunction, and psychological adjustment to altered body-image) is often unrecognized [2, 4, 5]. In spite of the importance, variability, and persistence of information and supportive care needs in BC patients, resources to meet these needs are very limited (e.g., support groups, survivorship programming, and patient navigation/information) [2, 4, 5].

In view of the increasing proportion of cancer survivors, centralization of advanced services in tertiary units, and patient desire to maximize local care for cancer surveillance, a shared care approach between urologists, oncologists, regional primary care physicians (PCPs) and advanced practice providers (APPs) might provide high-quality of survivorship care. Evidence supports surveillance models anchored in community-based survivorship care, which are non-inferior to oncologist-based care [6, 7]. Consequently, the American Society of Clinical Oncology (ASCO) recommends a shared care model between oncologists and PCPs for breast cancer survivorship; the level of shared care would be dependent upon the preferences of providers and survivors as well as available resources [8, 9]. Challenges to the community-based survivorship model include a lack of specialized experience in managing the complex needs of survivors [10, 11], scarcity of time to counsel patients about survivorship issues, complications of cancer treatments, psychosocial support, symptom management, and self-care [4, 5, 11, 12].

The Institute of Medicine (IOM), the National Comprehensive Cancer Network (NCCN), and American College of Surgeons Commission on Cancer (CoC) efforts to improve survivorship have led to major recommendations for enhancing cancer survivor’s care quality and follow-up care [5, 7]. One of these recommendations is the provision of Survivorship Care Plans (SCPs) to patients and their PCPs [13]. SCP is a personalized document summarizing patient’s diagnosis, treatment, surveillance and pertinent resources [13, 14]. The key elements of SCPs include prevention and detection of recurrent cancers, regular surveillance, interventions for long-term effects of the cancer and treatments, and coordination between PCP and specialists to ensure that survivor needs are met [13, 15, 16]. The IOM survivorship model emphasizes the value of a team approach of qualified specialists who collaborate to address patients’ unmet clinical and psychosocial supportive care needs during survivorship. This model recognizes existing gaps in clinical care as well as lack of multidisciplinary care approach contributing to patient existing needs and challenges in cancer survivorship.

Several SCPs have been formally developed for patients with childhood [17–19], breast [7, 20–23], gynecological [24] and colorectal cancers [25, 26], to help improve documentation and coordination of cancer treatment and survivorship care. A systematic review of these studies [27] has shown that the use of SCPs has resulted in high levels of cancer survivor satisfaction, high levels of survivor perception that SCPs enhanced communication between providers, and variable levels of distress [27, 28]. Increasing evidence points to the benefits and efficacy of survivorship care plans in reducing fatigue and distress, and improving long-term physical health and emotional well-being, physician-patient communication, patient experiences, and knowledge of recommended follow-up care [29, 30]. SCPs therefore provide an opportunity to engage survivors in their health care, while also capturing meaningful treatment-related outcomes to use as basis for making informed decisions [31]. However, research findings were not consistent in reporting these benefits as some studies failed to report these positive outcomes of the utility of SCPs in patients [21, 23, 24]. Reasons for the inconsistent research findings may be related to the use of different research methods, patient cohorts, or evaluation measures of patient’ outcomes.

BC patients represent a population likely to benefit from a disease-specific SCP (BC-SCP). In addition to the variability, persistence, and significant unmet informational and supportive care needs reported by this population across the disease trajectory, most of these patients are often treated regionally by specialists, creating an important need for communication with local physicians and other providers. We developed a disease-specific SCP to meet this need. While the use of generic SCPs among BC patients is largely unexplored, we hypothesize that a BC-SCP could improve patient-provider communication and bring a level of comprehensiveness and continuity to surveillance visits without adding burden to already busy provider clinics. We also believe that a BC-SCP will bring attention to the many unique unmet needs experienced by this population [4, 32, 33]. While we believe that SCPs play an essential role in improving communication and coordination between providers thus facilitating survivorship care among BC patients, availability of resources including clinic manpower, time, training and reimbursement, could be the potential challenges to foresee. In this study, we examined the acceptability and feasibility (i.e., the uptake and completion of the BC-SCP by clinical providers) of a BC-SCP we developed specifically for both non-muscle invasive bladder cancer (NMIBC) and muscle invasive bladder cancer (MIBC) patients.

## Methods

**Fig. 1:**
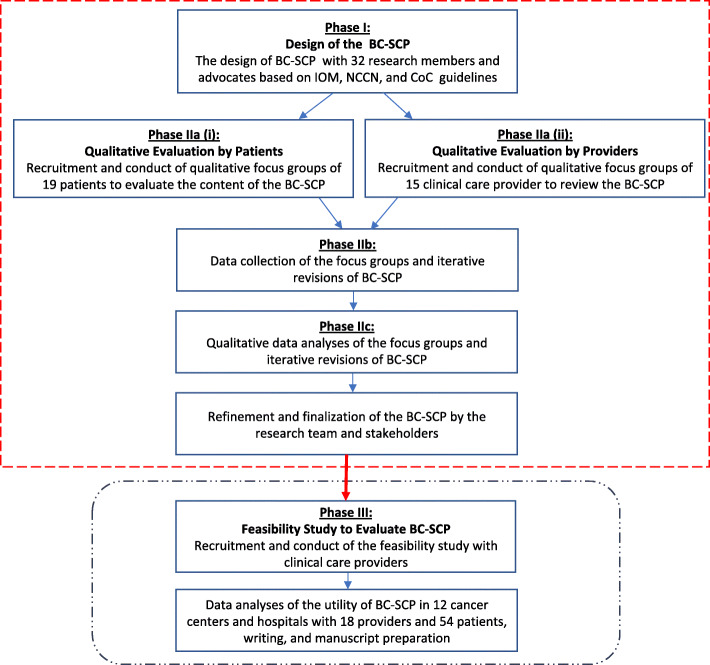
Phases of design, qualitative review, evolution of BC-SCP and feasibility study

This study was conducted between October 2011 and October 2012 and was approved by the Institutional Review Board (IRB) of the University of Michigan (the lead institution) and the participating institutions. Prior to the patient and provider focus groups (FG; Phase I) and provider usability study (Phase II), informed consent was obtained from the study participants. All study participants (Phase I and II) were consented before participation, were given a detailed description about the purpose of this project and ensured anonymity and confidentiality of their responses. FG participants received an honorarium of $100 for their time and transportation costs. The different phases of our work including the design, development, qualitative review leading to refinement of the content and evolution of our BC-SCP as well as the feasibility of it’s application into clinical practice is described below (Fig. 1).

### Phase I: the development of the BC-SCP

The BC-SCP (Additional file 1) was developed by two members of the research team - a urologist (CTL) and clinical psychologist (DML). In collaboration with The Survivorship Working Group of the Bladder Cancer Advocacy Network (BCAN-SWG) co-chaired by the same two members at the time of this study, the BC-SCP was iteratively reviewed and refined by all BCAN-SWG members including urologic oncologists, general urologists, medical oncologists, radiation oncologists, PCPs, physician extenders, nurse specialists, social scientists, BC survivors, and patient advocates (*N* = 32). The developers’ intent is to use the BC-SCP to summarize the treatment process of the patient and the aftercare that is needed for recovery, long-term surveillance, early detection of cancer recurrence, and overall health promotion [34].

Guidelines for SCPs recommended by the IOM, NCCN, and CoC informed the content, structure, and design of the BC-SCP [5, 7]. Following these guidelines, our BC-SCP (Additional file 1) incorporates sections for general and background information (Sections I and II), prior and planned treatment (Section III), and cancer surveillance (Section IV). An appendix to the care plan (Additional file 2) provides information regarding cancer prevention and health maintenance (Section A-I), “red-flag’ symptoms that should be reported (Section A-II), late effects of cancer and its treatment (Section A-III), and resources for providers and patients (Sections A-IV through A-VI). Lastly, a glossary is included in Section A-VII. Although healthcare providers are expected to complete and continue updating the BC-SCP, it is designed to also provide critical resource information to enhance patient-provider communication and to address common needs of BC patients (e.g., information about -ostomy nurses and support groups in the community). We designed BC-SCP for both low and high health literacy patients (Additional file 1) and added a Glossary to explain medical and clinical terms (Additional file 2).

### Phase II: qualitative evaluation of the BC-SCP by patients and clinical providers

#### Phase IIa: study participants

Following the revision of the BC-SCP based on BCAN-SWG reviews, we conducted two FGs with 19 BC patients clustered by gender (i.e., 11 male vs. 8 female groups) and two FGs with 7 physicians (e.g., urologists and oncologists) and 6 non-physician providers (e.g., physician assistants-PAs, nurse practitioners-NPs, social worker). The main goal of the FGs was to explore the acceptability of the content, design, structure, readability, and feasibility of the revised BC-SCP from both patient and provider perspectives. Time duration for each group ranged between 60 and 120 min. The providers (i.e., physician and non-physician) FGs were led by CTL and patient FGs were led by DML. Each FG met separately with the two facilitators (CTL and DML).

#### Phase IIb: data collection

Provider FG discussions were held over the phone and provided valuable input into 1) challenges faced by providers, 2) content of the BC-SCP, 3) the consolidation of health information from various provider sources, 4) treatment related complications, 5) psychosocial impact, 6) optimization of the surveillance plan, and 7) follow-up care. Additionally, they were asked about the quality of information regarding patient, care-giver, and provider resources. The facilitators then met with the male and female patient groups in person in a designated conference room space. Topics of interest that arose during the patient FGs included 1) patient awareness of disease, treatment and surveillance characteristics, 2) treatment complications, 3) unmet needs that emerge at diagnosis, after treatment, and during survivorship, and 4) a clear understanding of the BC-SCP content.

#### Phase IIc: data analysis

Following guidelines [35, 36], all FGs have been tape-recorded and transcribed verbatim. Two members of the research team (CTL and DML) met immediately after each FG to discuss critical points and notable quotes, and to identify topics that need more probing and clarifications in subsequent FGs. Tapes have been transcribed and interpreted through an iterative process of thematic content analysis using ATLAS.ti [37] by a third member of the research team (NEM) [35, 36]. Negotiated discussions (CTL, DML, HHG, and NEM) resolved discrepancies in emerging codes; findings revealed important characteristics and limitations of the BC-SCP. Results have been incorporated in further revisions of the BC-SCP’s content, structure, and readability.

### Phase III: feasibility study and survey evaluation of the BC-SCP with clinical providers

#### Phase IIIa: study participants

Urologic or medical oncologists, or APPs (including NP, PA) recruited new MIBC or NMIBC patients to be interviewed during standard clinic evaluations. Randomization method was not employed while selecting patients as the purpose of the study was to examine the feasibility of the SCP in clinical settings from the provider’s perspective using a convenient sample of patients. BC-SCP was completed on each patient. Data detailing the completion of the BC-SCP and the related encounter was captured. Each center was asked to complete a minimum of four SCPs, including two with MIBC and at least two with NMIBC. During clinical consultations, the BC-SCP was discussed with the patient; but SCPs were not handed over to patients, and patients were not asked to complete these SCPs, as the major goal of this study was to determine the feasibility of SCPs by providers in day to day medical practice in office based settings. Generic appendices containing patient resources and health information were provided to patients. Patient identifiers were not included in the BC-SCP.

#### Phase IIIb: data collection

An encounter data questionnaire developed by the research team for this study (Additional file 3) comprising of an 8-item survey using a seven-point Likert response scale (1–‘not at all’ to 7–‘very much’) was provided to participants to examine their evaluation of the BC-SCP and its feasibility. Single items assessed clarity of the BC-SCP, ease of use, ability to complete the BC-SCP during a clinical encounter, availability of resources to complete the BC-SCP, patient participation in completing the BC-SCP, use of BC-SCP to enhance patient-provider communication, and the degree of patient interest in receiving the patient resources and health information section of the BC-SCP. The survey-items also include questions assessing site characteristics (e.g., academic, private, Veteran Affair, other clinical settings), availability of EMR, provider characteristics (e.g., provider name, specialty, type, years in practice, and gender), availability of clinic support staff, willingness to complete the BC-SCP without reimbursement, number of rooms allocated to the clinic (e.g., general, consultation, and procedure rooms), and suggestions for improving the content of the BC-SCP.

#### Phase IIIc: data analysis

Both descriptive and comparative analyses were conducted to explore the feasibility and evaluation of the BC-SCP by providers (e.g., clarity of information). Statistical analyses described 1) site and provider characteristics, 2) completion time, and 3) provider evaluation of their actual use of the BC-SCP. Missing data was deleted automatically from the analyses. Imputation or missing value replacements were not used due to small sample size, and single item questions used to assess study outcomes. Comparative analyses were conducted on BC-SCP completion time stratified by patient clinico-pathologic parameters and provider specialty (urologist vs. other). Time to complete the BC-SCP was categorized into 1) ≤ 10 min, versus 2) > 10 min. Comparative analyses were conducted by means of the Chi-square test and confirmed by Fisher Exact test, a more conservative proportion-comparison test [38]. Significant differences were confirmed only with a two-tailed Fisher probability (*P*_Fisher_ < .05) [38]. Percentages were calculated to describe the frequencies within time groups (≤ 10 min and > 10 min). Quantitative descriptive analyses of data collected was carried out using SPSS analytical software.

## Study results

### Phase IIa (i): qualitative evaluation of the BC-SCP by patients

Challenges in BC clinical care and treatment outcomes reported by the patients included lack of full information about diagnosis, treatment, and outcomes; need for both written and visual educational tools about basic stoma care; change in urinary, sexual, and bowel function; nutrition; expectations regarding cystectomy and urinary diversion; and clinical support with psychological issues including altered body image, depression and anxiety. All patients reported that having BC-SCP would be helpful in updating the PCPs on recent results, treatments and follow-up plans; regular health promotion; and patient specific needs. Patients also felt that including detailed information regarding all the support resources available would be highly beneficial. Results of the qualitative assessments of both patients and providers informed further improvement in the content, structure, and delivery method of the BC-SCP (i.e., paper versions and electronic versions were made available for the usability testing of the BC-SCP).

### Phase IIa (ii): qualitative evaluation of the BC-SCP by clinical providers

Challenges in BC clinical care reported by the providers included patient education; treatment decision making, specifically in the context of neoadjuvant chemotherapy and urinary diversion; clinical care of patients failing intravesical therapies for NMIBC in the geriatric population; smoking cessation and psychological distress; and patient adherence to cancer surveillance. Although all providers reported high acceptability of the content, structure, and potential use of the BC-SCP, challenges and concerns about using the BC-SCP in the clinical setting was reported. These included the volume and breadth of information contained in the BC-SCP that might not be relevant to individual patients (i.e., depending on the specific cancer stage, treatment, and complications). This coupled with issues of scanning the BC-SCP into the media section in EPIC, manual data collection, and the inability to edit scanned information for future updates or changes in care, raised concerns in the feasibility of the paper version of the BC-SCP in busy clinics. All providers felt that an electronic version incorporated into the EMR would better facilitate care. Suggestions for improvement included the addition of contact details of stoma care nurses, pharmacists, physical therapists, and nutritionists as part of the care team involved in patient care. Reimbursement to account for provider time to complete the BC-SCP was another concern.

### Phase III: the feasibility of the BC-SCP

Twelve high-volume (i.e., > 50 cases/year) academic health-centers in the United States (US) and Canada, and one private practice group enrolled patients in this prospective clinical pilot. Of the 12 cancer centers and hospitals that participated in this study (Table 1), 90.9% had academic affiliations, and 72.7% had access to EMR. Of those with no active EMR, 66.7% had an EMR pending within 1 year. Of the 18 care providers who participated in this study, 81.2% were male, 36.4% reported having more than 20 years of experience in clinical practice (Table 2), 77.8% were urologists, 5.6% were medical oncologists and 16.6% were other care providers (i.e., NP, PA, medical assistants, social workers, residents, fellows, or student volunteers) (Table 3).

**Table 1:** Participating Sites

Site	Provider Type	Number of Patients
Icahn School of Medicine at Mount Sinai, NY, USA	Urologist	6
University of Michigan, MI, USA	Urologist	5
Sidney Kimmel Cancer Center at Jefferson, PA, USA	Medical Oncologist	5
University of Wisconsin School of Medicine and Public Health, WI, USA	Urologist	5
Penn State Hershey Medical Center, PA, USA	Urologist	5
McGill University Health Center, Quebec, CANADA	Urologist	5
University of Iowa, IA, USA	Urologist	5
University of Texas Southwestern Medical Center, TX, USA	Urologist	5
University of Chicago, IL, USA	Advanced Practice Providers	5
Urology Center of Colorado, CO, USA	Urologists	5
University of Florida, FL, USA	Urologist	2
National Cancer Institute, MD, USA	Urologist	1

**Table 2:** Site Characteristics

	n	%
No of Sites	12	–
Site Leader	12	–
Provider Gender		
Male	9	81.2
Female	2	18.8
Years in Practice		
< 10 Years	5	45.4
10–19 Years	2	18.2
20+ Years	4	36.4
Practice Setting		
Academic	10	90.9
Private	1	9.1
EMR^a^ active		
Yes	8	72.7
No	3	27.3
No active EMR		
EMR pending within 1 Year	2	66.7
NO pending EMR	1	33.3
Care Plan Preference		
Electronic	9	81.8
Hard Copy	2	18.2
**Available Support Staff**		
Nurse Practitioner (*n* = 10)		
None	4	40
1	5	50
> 1	1	10
Physician’s Assistant (*n* = 10)		
None	4	40
1	5	50
> 1	1	10
Nurse – other (*n* = 10)		
1	9	90
> 1	1	10
Medical Assistant (*n* = 10)		
None	1	10
1	7	70
> 1	2	20
Resident (*n* = 9)		
None	5	55.5
1	4	44.4
Fellow (*n* = 7)		
None	6	85.7
1	1	14.3
Student (*n* = 7)		
None	6	85.7
1	1	14.3
Volunteer (*n* = 8)		
None	7	87.5
1	1	12.5
Social Worker		
None	6	75.0
1	2	25.0
**Clinical Resources** (Mean ± SD)		
Number of clinic rooms	3 ± 1	n/a
General outpatient room	2.37 ± 1.10	n/a
Treatment / procedure room	1.18 ± 0.75	n/a
Consultation	1.0 ± 0.87	n/a

^a^Electronic Medical Record; *SD* standard deviation

**Table 3:** Care plan completion

Variable	n (%)
Number of Patients	54
Patient Gender (*n* = 47)	
Male	35 (74.5)
Female	12 (25.5)
Patient Race (*n* = 52)	
White	49 (94.2)
Black	2 (3.8)
Asian	0
Other - Hispanic	1 (1.9)
Patient Insurance Type (*n* = 51)	
Medicare	23 (45.1)
Medicaid	0
Private	19 (37.3)
VA	–
None	1 (2)
Other	8 (15.7)
Cancer Stage (*n* = 51)	
T0	4 (7.5)
T1	9 (16.7)
T2	19 (35.2)
T2N2	1 (1.9)
T3	4 (7.5)
T4	2 (3.7)
Ta	6 (11.1)
Tis	3 (5.6)
Tx	3 (5.6)
Provider Type Completing Care Plan (*n* = 18)	
Urologist	14 (77.7)
Medical Oncologist	1 (5.6)
Advanced Practice Providers (Nurse Practitioner / Physician’s Assistant)	2 (11.1)
Resident / Fellow	1 (5.6)
Completed Care Plan by Provider type (*n* = 54)	
Urologist	41 (75.9)
Medical Oncologist	5 (9.3)
Medical Assistant	–
Social Worker	–
Advanced Practice Providers (Nurse Practitioner / Physician’s Assistant)	7 (13)
Resident / Fellow	1 (1.9)
Student / Volunteer	–
Mean Completion Time: Mean (range)	12.2 min (2–25)
Completed in the presence of the patient? (*n* = 50)	
Yes	1 (2)
No	49 (98)
Where was care plan completed? (*n* = 49)	
General patient room	1 (2)
Staff room	15 (30.6)
Procedure / Treatment room	12 (24.5)
Consultation room	16 (32.7)
Outside of clinic	5 (10.2)
Did completion result in a higher billing code: (*n* = 46)	
Yes	0
No	46 (100)
Mean Number of	
New patients	4
Other patients	16

Fifty-nine BC patients were evaluated with the BC-SCP. Follow-up care plans were complete and valid for data-analysis in 54 (i.e., 92% completion rate) patients of whom 74.5% were male, 94.2% were Caucasians, 37.1% had MIBC, and 45.1% had Medicare as their primary medical insurance. Majority of the BC-SCPs were completed by urologists (75.9%) and 98% of BC-SCPs were completed before or after consultation with patients; 90% were completed within the confines of the clinic. The mean time to complete the SCP was 12.3 min (SD = 6; range: 2–25 min).

Frequencies of variables such as demographics of the patient, disease stage, insurance type, type of provider and their experience stratified within time groups (≤ 10 min and > 10 min) are shown in Table 4. Comparative analyses to examine differences in SCP completion time by clinical and demographic characteristics and provider type revealed no significant differences.

**Table 4:** Care plan completion time stratified by Clinico-pathologic parameters

Variable	Completed in ≤10 min (N = 18) N (%)	Completed in > 10 min (*N* = 23) N (%)	Chi-square	*p*-Value
Patient Age				
≤ 65 Years	2 (14.3%)	8 (42.1%)	2.83	.959
> 65 Years	12 (85.7%)	11 (57.9%)		
Patient Gender				
Male	12 (75%)	12 (66.7%)	2.38	.595
Female	4 (25%)	6 (33.3%)		
Race				
White	17 (94.4%)	20 (90.9%)	1.78	.673
Other	1 (5.6%)	2 (9.1%)		
Insurance Type				
Medicare	9 (52.9%)	10 (47.6%)	1.06	.744
Other	8 (47.1%)	11 (52.4%)		
Disease Stage2				
NMIBC	4 (23.5%)	8 (36.4%)	2.94	.086
MIBC	19 (76.5%)	14 (63.6%)		
Clinic Visits				
≤ 4 visits	8 (47.0%)	15 (71.4%)	1.99	.656
≥ 5 visits	9 (53.0%)	6 (28.6%)		
Provider Type				
Urologist	13 (72.2%)	17 (73.9%)	2.968	.397
Medical Oncologist	3 (16.7%)	2 (8.7%)		
Other	2 (11.1%)	4 (17.4%)		
Years in Practice				
≤10 Years	3 (33.3%)	6 (60%)	.533	.766
11–19 Years	3 (33.3%)	2 (20%)		
≥20 Years	3 (33.3%)	2 (20%)		

None of the care providers reported that completing the BC-SCPs resulted in higher billing codes. Provider experience with the BC-SCP is summarized in Table 5. Although the BC-SCP was clear and information to complete it was readily available, there was evidence that it may have had a negative impact on clinic flow. There was also some uncertainty expressed regarding the availability of clinic resources to complete BC-SCPs in all new BC patients. Since nearly all BC-SCPs were not completed in the presence of the patient, the patient was not active in its completion and the BC-SCP did not enhance dialogue with the provider.

**Table 5:** Evaluation of the Feasibility of the BC-SCP in Clinical Settings

Encounter Questions	Number of Encounter Responses	Median (mean) response; scale range: 1 (Not at all) - 7 (Very much)
Was the information that was requested clear?	48	6 (5.65)
Was it difficult to locate the requested information?	51	2 (3.10)
Is this format (assuming further revision) one you would consider using in your practice?	50	4 (4.04)
Did the care plan completion hamper clinic flow? If yes, please comment above.	34	3.5 (4.07)
Do you currently have ample clinic resources to complete survivorship care plans in all new patients?	50	4 (3.3)
Was the patient an engaged and active participant in the completion of the care plan?	35	2 (3)^a^
Did the care plan completion enhance the dialogue between you and the patient?	35	2 (2.89)^a^
Did the patient appear interested in receiving the appendix portion of the care plan?	33	3 (3.36)^a^

^a^98% of the care plans were filled without the presence of the patient

## Discussion

The major goals of this study are to examine the acceptability and feasibility of a BC specific SCP that we developed for patients to improve care and adherence to cancer surveillance. The current study qualitative and quantitative results demonstrated the high acceptability of the BC-SP by both patients and providers and completion rates of the BC-SCP in clinical settings by providers. Qualitative outcomes also confirmed some of the challenges experienced in BC care and in attempting to integrate BC-SCPs into clinical practice. These reported challenges by patients and providers confirmed our prior research findings on unmet needs of BC patients [2, 4] and their suggestions for BC-SCP improvement were integrated in iterative revisions of the content and design of the BC-SCP and its appendices (e.g., online BC support groups; ostomy nurses in the community).

Phase III feasibility survey data revealed that although providers had access to a clear and concise BC-SCP that could be completed in the clinic, its use did not enhance patient engagement or the patient-physician interaction because of time constraints. This is likely to reduce expected patient benefits from BC-SCP discussions. Providers had a mean number of three clinic rooms and one consultation room in which to see patients. Rather than slow room turnover, providers opted to complete the BC-SCP away from patients, in their own personal time, to avoid interruption to clinic flow. Although a nurse was available within most practice environments, support staff were not plentiful as evidenced by ≥40% of practices without an APP, > 55% without a specialty resident, and > 85% without a clinical fellow. Ultimately, given the increased time for SCP completion, without a clear increase in billing, the SCP completion by genitourinary specialists does not seem sustainable.

Multiple studies in other cancer populations have cited lack of staff, SCP templates, time to complete SCP, training and reimbursement, and the time necessary to obtain information required to create an SCP, as barriers for SCP use [39]. Our results suggest that the majority of the BC-SCPs in this study were completed by the patients’ providers devoting a mean of 12.3 min to this task. As health systems in the US continue to mobilize efforts to comply with the CoC recommendations to provide treatment summary and SCPs to eligible patients [39–41], it will be incumbent upon Cancer Center leaders to provide appropriate support staff to achieve this goal within working time periods. As the emphasis on clinical productivity and patient throughput grows across academic and private health-centers, providers could potentially be constrained by time, which might limit the use of the BC-SCP by providers during consultations as well as sharing and discussing the BC-SCP with patients during the clinical encounter. This necessitates the expansion of work force (e.g., the involvement of trained clinical support personnel) or provision of extra billable time to ensure optimal use of the BC-SCP in clinical settings. Moreover a successful survivorship program requires adoption of a multidisciplinary approach; a dedicated survivorship program, possibly run by an APP and mental health providers trained in BC care and surveillance with consultation by a physician provider could accommodate and make valuable the use of a SCP. However, the provision of resources and funding remain a major challenge to establish the necessary infrastructure required for such a dedicated approach.

Survivorship care planning may be an important driver of surveillance and follow-up for cancer survivors; however, over a decade into the proposed SCP by IOM, robust evidence supporting the large-scale implementation of care plans amongst cancer survivors or to abandon SCPs altogether is lacking [42]. Although SCPs have largely been tested in colorectal, breast, ovarian and childhood cancers, they haven’t gained much momentum in the urologic community despite the existence of guidelines on surveillance of urologic cancers treated at various stages. This was part of the incentive to consider the current study. We believe that incorporating a disease-specific SCP, developed via an iterative process with input from patients at multiple points, into active practice settings would truly provide a holistic approach to patient care. However, observed challenges in the actual use of SCP remain to be addressed.

In sum, BC-SCPs are necessary to improve patient care and outcomes. Survivors treated for NMIBC need regular cystoscopic surveillance to detect recurrence. MIBC survivors treated with radical cystectomy or radical radiotherapy need frequent monitoring to detect metastatic disease as well as follow-up for changes in urinary, bowel, metabolic, sexual disturbances, and psychological and emotional consequences. Gilbert et al. suggested that care of urologic cancer survivors may be improved by i) the widespread implementation and application of SCPs, ii) adoption of evidence-based surveillance practices in the follow-up of cancer survivors, and iii) the development of disease-specific and institution-based survivorship clinics to systematically improve survivorship care [42]. Moreover, Gilbert suggested that the use of a standardized SCP can facilitate communication between providers, serve as a guide for follow-up care, and help coordinate disease and health surveillance [43].

Our study has limitations. First, the survey population is limited to a very small number of providers, albeit from various institutions across the country with a total of 54 patients information included. Because of the small survey sample size, we did not examine potential interactions between site and provider characteristics on the SCP evaluation or completion time. Second, a majority of the providers in our study were urologists with a small proportion of the providers being medical oncologists and NPs. Another limitation of our study is the absence of the views of PCPs regarding the content and usefulness of the BC-SCP. The primary focus of our project at this point, however, was to assess the acceptability and feasibility of implementing the BC-SCP in clinical practice of the specialty providers involved directly in cancer care. The usefulness of the actual content and its validity would need to be assessed in future studies. Finally, just over half of the BC-SCPs were completed in the consultation room and procedure/treatment room, while the remainder were completed elsewhere. It might be interesting to see if there would be any difference to the quality of information and timing for completion if the SCPs were to be completed during the actual patient-physician encounter, to obtain views of primary care physicians on the ease of use of SCPs in primary care settings to improve follow-up care, and to incorporate SCPs into EMR; future studies could potentially evaluate this. Larger randomized controlled studies on innovative models of SCP, the impact of these SCPs on oncological, psychosocial and resource outcomes are required. Despite these limitations however, our findings have implications for utilizing and implementation of the BC-SCP that we have developed.

As the use of SCPs increases, it is important to enhance research comparing the effectiveness, usefulness, impactful elements of SCP and the timing in which they should be delivered [44]. Further studies assessing a range of outcomes in various survivor populations and using different outcome measures are required [45] to fully understand the efficacy of SCPs.

## Conclusion

A BC specific SCP has clinical relevance but there are significant challenges for providers’ use of the SCP with BC patients in an active practice setting in specialty clinics.. Urologic specialists can complete BC-SCPs in a timely manner but will likely choose to do this outside of the patient encounter which may limit the impact of the SCP on patients. Increased clinic resources and personnel may be leveraged to complete BC-SCPs on a greater scale in an effort to comply with CoC guidelines but to also enhance patient experience. Integration of the BC-SCP within the EMR may increase the efficiency of BC-SCP implementation and the likelihood of integrating it into regular communications with a range of healthcare providers.

## Supplementary information

**Additional file 1.** The Bladder Cancer Survivorship Care Plan (BC-SCP). This file includes information pertaining to general information, background information, treatment plan and summary, follow-up care.

**Additional file 2.** Resource Guide. This file incorporates preventive / health maintenance information, alarm symptoms and signs to watch out for, potential late-effects of cancer, benefit resources, resources for health care providers, resources for bladder cancer survivors and families.

**Additional file 3.** Encounter Data Sheet. This file includes information pertaining to the logistics of completing the BC-SCP such as the provider type and the time taken to complete the BC-SCP, 8 item-survey with 7-point Likert response scale.

## Data Availability

The datasets generated and/or analyzed during the current study are not publicly available due to most of our data being qualitative information and analysis involving FGs; but are available from the corresponding author on reasonable request. Data regarding participating sites, data analysis, the SCP designed and used, resource guide and encounter data sheet are all included as supplementary files in this submission.

## Abbreviation

APP: Advanced Practice Providers
BC: Bladder Cancer
BC-SCP: Bladder Cancer Survivorship Care Plan
BCAN: Bladder Cancer Advocacy Network
CoC: Commission on Cancer
EMR: Electronic Medical Record
FG: Focus Group
IOM: Institute of Medicine
MIBC: Muscle Invasive Bladder Cancer
NCCN: National Comprehensive Cancer Network
NMIBC: Non Muscle Invasive Bladder Cancer
NP: Nurse Practitioner
PA: Physician Assistant
QoL: Quality of Life
SCP: Survivorship Care Plans
SD: Standard Deviation
SWG: Survivorship Working Group
