# Supplementary material for: Development and evaluation of a bladder Cancer specific survivorship care plan by patients and clinical care providers: a multi-methods approach
Source: BMC Health Serv Res. 2020 Jul 24;20:686. doi: 10.1186/s12913-020-05533-7 (PMC7379822; doi:10.1186/s12913-020-05533-7)
Supplement: Supplementary file 1 — Additional file 1. The Bladder Cancer Survivorship Care Plan (BC-SCP). This file includes information pertaining to general information, background information, treatment plan and summary, follow-up care. [file 12913_2020_5533_MOESM1_ESM.pdf]

Bladder Cancer Survivorship Care Plan

This Survivorship Care Plan will facilitate cancer care ***following*** active treatment. It may include important contact information, a treatment summary, recommendations for follow-up care testing, a directory of support services and resources, and other information.

Bladder Cancer Advocacy Network
BCAN®
Leading the way to awareness and a cure

Prepared by:
Date Prepared:

I. General Information

Survivor Information

Name	
Gender	
ID	
Phone	Preferred contact
Date of birth __ __ / __ __ / __ __ __ __	
Age at diagnosis	
Support contact	

Care Team

**Doctor**	**Name**	**Contact Information**
Urologic Oncologist (specialist)		
General Urologist		
Medical Oncologist		
Radiation Oncologist		
Primary Care Physician		
Nurse/Advanced Practice Nurse		
Mental Health/Social Worker		
Nutritionist/Dietician		
Wound/Ostomy Nurse		
Other (Therapist/Pharmacist/etc.)		

Important caution: This is a summary document whose purpose is to review the highlights of the cancer treatment plan for this patient. This does not replace information available in the medical record, a complete medical history provided by the patient, examination and diagnostic information, or educational materials that describe strategies for coping with cancer in detail. Both medical science and an individual's health care needs change, and therefore this document is current only as of the date of preparation. This summary document does not prescribe or recommend any particular medical treatment or care for bladder cancer or any other disease and does not substitute for the independent medical judgment of the treating professional.

51120 11/11 CHR

II. Background Information

Initial bladder cancer diagnosis date	
Tumor histology (type & date)	
Highest Tumor grade	
Clinical stage at diagnosis (TNM)	
Major Comorbid Conditions/Other Cancer History	
Additional Comments	

Bladder Cancer Recurrence	Dates	Body Location	Treatment	Dates of treatments
	__ __/ __ __/ __ __ __ __			__ __/ __ __/ __ __ __ __
	__ __/ __ __/ __ __ __ __			__ __/ __ __/ __ __ __ __
	__ __/ __ __/ __ __ __ __			__ __/ __ __/ __ __ __ __

III. Treatment Plan & Summary

Tumor Resections (TURBT)	Dates	Clinical Stage (TNM)	Location
	__ __/ __ __/ __ __ __ __		
	__ __/ __ __/ __ __ __ __		
	__ __/ __ __/ __ __ __ __		

Intravesical Agents	# Treatments	Dates of treatments	Dose
		__ __/ __ __/ __ __ __ __--__ __/ __ __/ __ __ __ __	
		__ __/ __ __/ __ __ __ __--__ __/ __ __/ __ __ __ __	
		__ __/ __ __/ __ __ __ __--__ __/ __ __/ __ __ __ __	

Radical (Major) Surgery or Radiation for Urinary Tract	Date	Tumor Stage (TNM)	Location
	__ __/ __ __/ __ __ __ __		

Systemic Chemotherapy Agents (Drugs)	# Cycles	Start Dates	End Dates
		__ __/ __ __/ __ __ __ __	__ __/ __ __/ __ __ __ __
		__ __/ __ __/ __ __ __ __	__ __/ __ __/ __ __ __ __
		__ __/ __ __/ __ __ __ __	__ __/ __ __/ __ __ __ __

Clinical Trials	Treatment/ Intervention	Dates	Hospital
		__ __/ __ __/ __ __ __ __	
		__ __/ __ __/ __ __ __ __	
		__ __/ __ __/ __ __ __ __	

Supportive treatment to improve quality of life	Dates
	__ __/ __ __/ __ __ __ __
	__ __/ __ __/ __ __ __ __
	__ __/ __ __/ __ __ __ __

IV. Follow-up Care Without Recurrence

The specific tests and the frequency of follow-up outlined below are general recommendations based upon NCCN guidelines and expert consensus. Their application may vary from one physician to another depending on the specifics of each patient and their tumor. The patient should discuss the details of cancer surveillance with his or her own physician. Recurrent cancer will require a deviation from the schedule below to consider additional treatments.

TURBT (Bladder Tumor Resection) or Partial Cystectomy (Partial Bladder Removal)

	Year 1	Year 2	Years 3-5	Year 5+
Cystoscopy[1]				
**Low Risk:** LG[2] Ta	Every 3-6 months	3-12 months	6-12 months	As Indicated
**Med Risk:** Large, Multiple, Recurrent LG[2] Ta	Every 3 months	3 months	6 months	Annually
**High Risk:** HG[2] Ta, CIS[2], ≥T1	Every 3 months	3 months	6 months	Annually
Medical history and Focused Physical Exam	With cystoscopy	With cystoscopy	With cystoscopy	With cystoscopy
Urine cytology or other marker	With cystoscopy	With cystoscopy	With cystoscopy	With cystoscopy
Upper Tract Imaging[3]				
**Low Risk:** LG[2] Ta	With recurrence			
**Med Risk:** Large, Multiple, Recurrent LG[2] Ta	Every 1-2 years			
**High Risk:** HG[2] Ta, CIS[2], ≥T1	Every 1-2 years			

[1]There is an accepted range for the frequency of cystoscopy based on the clinical situation.
[2]LG: low grade; HG: high grade; CIS: carcinoma in situ
[3]Upper tract imaging of the kidneys and ureters can be accomplished with a CT scan, MRI, or renal ultrasound.

Radical Cystectomy (Complete Bladder Removal)

	Year 1	Year 2	Years 3-5	Year 6+
Medical History and Physical Exam				
**Low Risk:** pT0-pT1	Every 3-6 months	6 months	Annually	Every 1-2 years
**Med Risk:** pT2	Every 3 months	6 months	Annually	Every 1-2 years
**High Risk:** ≥pT3 or N+	Every 3 months	6 months	6-12 months	Annually
CT or MRI (abdomen and pelvis)				
**Low Risk:** pT0-pT1	Annually	Annually	Annually	Renal
**Med Risk:** pT2	Annually	Annually	Annually	Ultrasound
**High Risk:** ≥pT3 or N+	Every 3-6 months	3-6 months	Annually	Every 2 years
Chest Imaging				
**Low/Med. Risk:** chest x-ray	Every 3-6 months	6 months	Annually	As Indicated
**High Risk:** chest CT	Every 3-6 months	3-6 months	6-12 months	As Indicated
Urine Cytology or Other Marker	As Indicated	As Indicated	As Indicated	As Indicated
Urethral Wash (Cutaneous Diversion)				
**Low Risk:** No CIS & no urethral cancer	If symptomatic	If symptomatic	If symptomatic	If symptomatic
**Med Risk:** bladder CIS & no urethral cancer	Every 6-12 months	6-12 months	If symptomatic	If symptomatic
**High Risk:** bladder CIS with urethral cancer	Every 3-6 months	6 months	6-12 months	If symptomatic
Serum Electrolytes, BUN/Creatinine[4]	Each visit	Each visit	Each visit	Each visit

[4]Additional laboratory studies such as CBC (blood counts), vitamin B-12 (for patients with a continent diversion), and liver function tests may be required, as indicated.

Urinary Diversion

	Year 1	Years 2-5
Urostomy (Stoma)	Months 1,2,3,6: Stoma (size, shape, color, texture)<br>Peristomal skin integrity<br>Appliance System (leakage, wear time, ease of use)	Each visit
Colon Pouch	Months 1, 3, 6, 12: Stoma (size, color)/<br>Urinary leakage/Catheterization	Each visit
Neobladder	Months 1, 3, 6, 12: Urine storage (leakage, mucous)<br>Urinary emptying (frequency, flow, mucous)	Each visit

Within the first 3 months after cystectomy, upper tract imaging should be obtained to identify subclinical ureteral stricture. Likewise, patients should be questioned about symptomatic urinary tract infection at each visit.
