# Supplementary material for: Development and evaluation of a bladder Cancer specific survivorship care plan by patients and clinical care providers: a multi-methods approach
Source: BMC Health Serv Res. 2020 Jul 24;20:686. doi: 10.1186/s12913-020-05533-7 (PMC7379822; doi:10.1186/s12913-020-05533-7)
Supplement: Supplementary file 2 — Additional file 2. Resource Guide. This file incorporates preventive / health maintenance information, alarm symptoms and signs to watch out for, potential late-effects of cancer, benefit resources, resources for health care providers, resources for bladder cancer survivors and families. [file 12913_2020_5533_MOESM2_ESM.pdf]

APPENDICIES

A-I. Preventive Measures

Health Maintenance	Source
Bone health	Participate in regular weight bearing exercise (walking, strength training). Take recommended amounts of calcium and vitamin D. Talk with your provider about a vitamin D blood test. Monitor bone density.
Cancer screening	Annual physical exam with primary care provider.<br>Stay current on routine cancer screenings (gynecology, breast, colon, prostate, skin checks).
Cholesterol monitoring/ management	Annual physical exam with primary care provider
Dental care	Biannual dental exam
Diabetic screening/ management	Annual physical exam with primary care provider
Diet	American Institute for Cancer Research (AICR) **www.aicr.org/**<br>Make fruit and vegetables the basis for most of your meals and snacks.<br>Consume healthy fats (olive oil, nuts) and avoid processed foods. Eat smaller portions of animal fats and proteins or eat them less often. Ask for referral to nutritionist/dietician.
Exercise/Weight management	Adopt a physically active lifestyle. Do at least 30-45 minutes of moderate to vigorous activity at least 5 days/week. Request a referral for physical therapy for help setting goals and dealing with physical limitations.
Hypertension control	Annual physical exam with primary care provider.
Mental health	Seek support as soon as you can after you receive your cancer diagnosis. There are a number of local in-person and online support groups, as well as the love and support of your partner, family, and friends. If you notice long periods of feeling hopeless or helpless about your situation or changes in sleeping patterns or energy level, consult your primary care provider for mental health resources in your community or use the resources below to find someone who can help you through the challenges of living through cancer.
Smoking cessation	Many cancer centers and other medical facilities offer smoking cessation programs. The most effective are those that combine behavioral counseling and medication to reduce the physiological craving for cigarettes, and other tobacco products. Survivors should be offered referrals to help quit smoking at the time of diagnosis.
Vaccines	Annual physical exam with primary care provider.

51120b 11/11 CHR

A-II. Symptoms to Watch For

Patient should report these signs and symptoms if persistent:

- Abdominal (stomach) pain
- Anxiety
- Back pain
- Blood in the urine (hematuria)
- Blood or other drainage from the vagina (in women) or the penis (in men)
- Bone pain or fractures
- Chest pain
- Concerns about cancer recurrence
- Depression
- Leg swelling
- Loss of appetite
- Nausea
- New lumps in the groin area
- Relationship problems related to cancer and its after effects
- Sexual difficulties, including low desire, trouble with erections, problems with vaginal dryness, delay or lack of orgasm, etc.
- Shortness of breath or difficulty breathing
- Skin breakdown around your stoma
- Urine leakage from your urostomy system
- Unexplained weakness or fatigue
- Unplanned weight loss
- Urinary difficulties, including urgent or frequent urination, or loss of urine control (incontinence)
- Vomiting

A-III. Potential Late-Effects of Cancer

You may experience the following effects after cancer treatment:

- **Intravesical Therapy (Bladder Treatments):** Urgent or frequent need to urinate, painful urination, urinary leakage, psychological distress
- **Bladder Resection (TURBT):** Urgent or frequent need to urinate, painful urination, urinary leakage; urethral stricture or scarring, psychological distress
- **Partial Cystectomy (Partial Bladder Removal)** Urgent or frequent need to urinate, psychological distress
- **Radical Cystectomy (Bladder Removal)**
  Urinary problems: urine leakage; urinary tract infection, scarring, or obstruction; narrowing of the stoma (for patients with a stoma); bladder stones or trouble urinating (for patients with a neobladder); kidney stones, loss of kidney function
  Bowel problems: diarrhea, constipation, or bowel obstruction
  Other: sexual dysfunction, psychological distress, electrolyte abnormalities (changes in the body salts); abdominal hernia
- **Chemotherapy** Neuropathy, cognitive dysfunction, psychological distress, fatigue, metabolic syndrome (elevated cholesterol, elevated glucose, weight gain)
- **Radiation** Fatigue, scarring, bladder or rectal irritation and bleeding, bowel obstruction, psychological distress

A-IV. Selected Benefit Resources

Agency	Contact Informant	Services
Government Benefits	www.benefits.gov<br>1-800-FED-INFO	Health coverage, including Medicaid
Medicare	www.medicare.gov<br>1-800-MEDICARE	Health coverage for seniors
Social Security	http://www.socialsecurity.gov/<br>1-800-772-1213	Benefits for seniors and those with disabilities
Partnership for Prescription Assistance	http://www.pparx.org/<br>1-888-477-2669 (888-4PPA-NOW)	Assistance obtaining prescription drugs
RX Assist's Patient Assistance Program Center	http://www.rxassist.org<br>E-mail: info@rxassist.org	Searchable database (drug name/ company prescription assistance program)
Employee Assistance Program (EAP)	Employee contact with the EAP is kept private. To learn more, ask your employer about your company's EAP. (This service is offered, for the most part, through large employer groups.)	EAPs help employees and their families deal with issues such as:<br>• Short and long-term disability<br>• Personal and emotional concerns<br>• Legal and financial advice

Bladder Cancer Advocacy Network:

BCAN–the Bladder Cancer Advocacy Network–is the first national advocacy organization dedicated to increasing public awareness about bladder cancer; to advancing bladder cancer research; and to providing educational and support services for the bladder cancer community. Founded in May 2005, BCAN is a cooperative effort among bladder cancer survivors, their families and caregivers, and the medical community. A survivorship tool kit is currently under development for health care providers.
http://www.bcan.org/

American Cancer Society (ACS) Guidelines on Nutrition and Physical Activity for Cancer Prevention

Updated every five years, this document is a short version of the ACS Nutrition and Physical Activity Guidelines. It includes how to maintain a healthy weight and how to stay active.
http://www.cancer.org/Healthy/index

From Cancer Patient to Cancer Survivor: Lost in Transition Report Recommendations–November 2005

The recommendations in this report, taken from the Institute of Medicine's report, From Cancer Patient to Cancer Survivor: Lost in Transition, are directed to cancer patients and their advocates, health care providers and their leadership, health insurers and plans, employers, research sponsors, and the public and their elected representatives.
https://www.nap.edu/catalog/11468/from-cancer-patient-to-cancer-survivor-lost-in-transition

Cancer Survivorship Care Planning– November 2005

A report by the Institute of Medicine based on From Cancer Patient to Cancer Survivor: Lost in Translation, 2006, details the elements of a survivorship care plan as well as frequent questions Survivors may ask.
https://www.nap.edu/catalog/11468/from-cancer-patient-to-cancer-survivor-lost-in-transition

Cancer Care for the Whole Patient: Meeting Psychosocial Health Needs–October 2007

The report by the Institute of Medicine studies the delivery of psychosocial services to cancer patients and their families and identifies ways to improve it.
https://www.nap.edu/catalog/11993/cancer-care-for-the-whole-patient-meeting-psychosocial-health-needs

National Cancer Institute: Office of Survivorship

The mission of the Office of Cancer Survivorship (OCS) is to enhance the quality and length of survival of all persons diagnosed with cancer and to minimize or stabilize adverse effects experienced during cancer survivorship. Resources for physicians include clinical practice follow-up guidelines, management of late-term effects of cancer treatment, and information regarding supportive care.
https://cancercontrol.cancer.gov/ocs

American Psychosocial Oncology Society (APOS)

APOS publishes a pocket guide to psychosocial care for cancer patients, survivors, and their families.
http://www.apos-society.org

Oncology Nursing Society (ONS)

ONS publishes a number of useful references for nurses and others providing care to cancer patients and survivors, particularly with regard to symptom management. They also offer regular opportunities for continuing education.
www.ons.org

Association of Oncology Social Work (AOSW)

AOSW provides a wide variety of resources for social workers who provide care to cancer patients, survivors, and families. AOSW also has resources to assist with patient navigation.
www.aosw.org

The National Hospice and Palliative Care Organization (NHPCO)

The NHPCO is the largest nonprofit membership organization representing hospice and palliative care programs and professionals in the United States. The organization is committed to improving end of life care and expanding access to hospice care with the goal of profoundly enhancing quality of life for people dying in America and their loved ones. The site includes a searchable database to find local hospice services.
www.nhpco.org

A-VI. Resources for Bladder Cancer Survivors and their Families

Area of Interest	Organization	Contact Information	Services
Bladder Cancer Information/Treatment	**Bladder Cancer Advocacy Network (BCAN)**	http://www.bcan.org/	The first national advocacy organization for bladder cancer survivors; provides educational and support services for the bladder cancer community
Cancer Survivorship	**Institute of Medicine: From Cancer Patient to Cancer Survivor: Lost in Transition - video**	https://www.youtube.com/watch?v=YhuqWM3dNAw	This short film by the Institute of Medicine features the stories of cancer survivors and supports the need for a Survivorship Care Plan.
	**National Cancer Institute: The Facing Forward Series**	https://www.cancer.gov/publications/patient-education/facing-forward	A series of booklets designed to educate cancer survivors, family members, and health care providers about the challenges associated with life after cancer treatment.
Chemotherapy (Systemic)	**Cancer.Net™**	https://www.cancer.net/survivorship	Patient education
Complementary Therapy	**National Center for Complementary and Alternative Medicine (NCCAM)**	http://nccam.nih.gov<br>1-888-644-6226<br>email to info@nccam.nih.gov	The federal government's lead agency for scientific research on complementary and alternative medicine. Provides patient education.
Depression/Anxiety	**American Psychosocial Oncology Society (APOS)**	http://www.apos-society.org<br>Help line (1-866-276-7443)	Locate a mental health provider in your area.
	**CancerCare**	http://www.cancercare.org/<br>1-800-813-HOPE (4673)	Gives free support services to those affected by cancer; programs include counseling, education, financial and practical help.
General Cancer Information and Advocacy	**American Cancer Society (ACS)**	http://www.cancer.org/Treatment/Support ProgramsServices/index<br>(support services)<br>http://csn.cancer.org/<br>(Cancer Survivors Network)	Wide variety of services and supportive care for cancer survivors and their families.
	**Cancer.Net™**	http://www.cancer.net/portal/site/patient	Detailed patient information website of the American Society of Clinical Oncology® (ASCO); helps patients and families make informed health-care decisions.
	**Cancer Support Community (CSC)**	www.cancersupportcommunity.org<br>1-888-793-WELL<br>email: help@cancersupportcommunity.org	Global nonprofit group that gives support, education and hope to people with cancer and their loved ones.
	**Lance Armstrong Foundation - LIVESTRONG**	www.livestrong.org<br>1-855-220-7777	Educational articles and videos
	**National Coalition for Cancer Survivorship (NCCS)**	www.canceradvocacy.org<br>1-877-622-7937	Advocates for quality cancer care; provides the Cancer Survival Toolbox to survivors, caregivers and clinicians at no cost.
Incontinence, Urine Control, Supplies and Concerns	**Wound Ostomy and Continence Nurses Society (WOCN)**	www.wocn.org<br>1-888-224-9626	Nurse experts who specialize in the care of people with wounds, ostomies and incontinence issues.
	**Dr. Leonard's Healthcare Catalog**	www.drleonards.com<br>1-800-785-0880<br>www.nationalincontinence.com<br>1-800-998-1745	On-line healthcare catalogue with incontinence products.<br>On-line incontinence supplies

A-VI. Resources for Bladder Cancer Survivors and their Families

Area of Interest	Organization	Contact Information	Services
Nutrition Before and after Treatment	**ACS Guidelines on Nutrition and Physical Activity for Cancer Prevention**	www.cancer.org/Healthy/index	How to maintain a healthy weight and stay active; updated every five years.
	**CSC (see above)**	www.cancersupportcommunity.org<br>1-888-793-WELL	Provides information on nutrition and exercise programs, as well as stress reduction classes.
Sexual Dysfunction	**American Association of Sex Educators, Counselors, and Therapists (AASECT)**	http://www.aasect.org	AASECT provides information and referrals for persons having difficulty with sexual issues from a variety of causes including cancer and chronic disease.
Stomal Supplies and Concerns	**United Ostomy Associations of America (UOAA)**	http://www.ostomy.org/home.html<br>1-800-826-0826	Provides support, information and advocacy to persons with a stoma and their caregivers
	**Wound Ostomy and Continence Nurses Society (WOCN)**	www.wocn.org or 1-888-224-9626	Nurse experts who specialize in the care of people with wounds, ostomies and incontinence issues.
	**C3Life.com**	www.c3life.com/ostomy	Online moderated forum for people with ostomies
	**Coloplast**	www.coloplast.com/ostomycare<br>on line interactive user guide, sample line	Ostomy equipment
	**ConvaTec**	www.convatec.com<br>1-800-422-8811<br>online information, help and sample line	Ostomy equipment
	**Cymed**	http://cymedostomy.com/home.html<br>1-800- 582-0707<br>Samples available	Ostomy equipment
	**Hollister**	www.hollister.com<br>1-888-740-8999<br>online learning center, sample line	Ostomy equipment

A-VII. Glossary of Terms

Adjuvant – Treatment offered after an initial treatment for cancer, especially to reduce secondary tumor formation.

Anxiety – A feeling of worry, nervousness, or unease that may be related to one's health situation.

Bacteruria – The presence of bacteria in the urine that can cause infection.

BCG (Bacille Calmette-Guérin) – A treatment for nonmuscle-invasive bladder cancer that is put into the bladder to prevent reccurrence of the cancer. BCG is considered immunotherapy – a treatment that stimulates a response from the immune system.

Bowel Obstruction – A blockage that prevents food passing from the stomach through the lower intestine and out the body. The blockage can occur after the removal of the bladder.

Cancer – Cancer is the general name for a group of more than 100 diseases in which cells in a part of the body begin to grow out of control. Although there are many kinds of cancer, they all start because abnormal cells grow out of control. Untreated cancers can cause serious illness and even death.

Cancer Stage – Cancer severity is described using a standard system called staging. Since the system is standardized, different doctors can talk about a patient's cancer and understand exactly what the patient's status is. The TNM Classification of Malignant Tumors (TNM) is a cancer staging system that describes the extent of cancer in a patient's body. T describes the size of the tumor and whether it has invaded nearby tissue, N describes whether the cancer has entered the lymph nodes near where the cancer was found, and M describes whether the cancer has spread from the original body part to others.

Cancer Survivor – A cancer survivor is a person affected by cancer. Different people define being a survivor in different ways. Some feel that being a cancer survivor begins when the person is diagnosed with cancer. Others believe that a survivor is someone who has completed cancer treatment and is now trying to return to their life as it was before cancer.

Chemotherapy – Physicians sometimes treat cancer with special medicines designed to kill just the cancer cells. Chemotherapy can sometimes be given in addition to other treatments like radiation or surgery or may be given when cancer returns after an earlier treatment.

Cognitive Dysfunction – Some cancer treatments can cause changes in thinking patterns or memory. These changes in mental functioning can sometimes be hard to diagnose in older patients who may have some mental slowing due to the natural aging process.

Comorbid – People can have more than one disease at a time. When a physician is treating a person for one disease and another one is present, the second disease is called a comorbid condition.

Complication – Treatments for disease can sometimes cause other medical problems. For example, treatments for bladder cancer may sometimes cause incontinence or sexual dysfunction.

Conduit – (See Stoma Below)

Cystectomy – Surgical removal of the bladder. In men, the prostate and seminal vesicles may also be removed. In women, a hysterectomy (removal of the uterus) may also be performed.

Cystoscopy – An examination usually performed by a urologist. A camera is inserted into the urethra (the opening where urine comes out) to find out whether there are cancer cells in the bladder.

Depression – Depression is a strong mood involving sadness, discouragement, despair, or hopelessness that lasts for weeks, months, or even longer. Depression can be related to stressful events like a cancer diagnosis, living with side effects of cancer treatment, concerns about death and dying, or many other reasons. Sometimes depression can go away without treatment but usually people living with depression need help from a counselor or other person they can talk to about their concerns. Sometimes a medication for depression can be helpful.

Erectile Dysfunction (ED) – Problems with maintaining an erect penis needed to have sexual activity. Erectile dysfunction can be complete or partial. Treatments for ED include pills, injections, and others. Having ED can cause a man distress and reduce his confidence in his ability to successfully satisfy himself and his partner. Some men may benefit from seeing a counselor to help reduce their distress about ED and face other problems that the man and his partner may have in their relationship.

Hematuria – Blood in the urine. While blood in the urine can be caused by many things, it is the main symptom of bladder cancer. A person who sees blood in their urine should always consult a physician to find out the cause and be treated.

Incontinence – Incontinence is uncontrolled leakage of urine. Incontinence can be described by how often a person leaks urine and what led up to the leaking (example: leaking urine when you sneeze or cough).

Intravesical –This term refers to the inside of the bladder. Treatments for bladder cancer that are put into the bladder are referred to as intravesical therapy (example: see BCG above).

A-VII. Glossary of Terms

Neoadjuvant – Treatment that is provided before the main treatment. For example, in treating bladder cancer, some patients will have neoadjuvant chemotherapy before the surgical removal of their bladder.

Neuropathy – Damage to nerves that causes pain, usually in the hands and feet, but can occur other places.

Oncologist – A physician trained to treat cancer.

- **Medical Oncologist –** A physician who primarily uses medication to treat cancer (example: chemotherapy).
- **Urologic Oncologist –** A physician who primarily uses surgery to treat cancer of the urinary system.
- **Radiation Oncologist –** A physician who primarily uses radiation (energy that can kill cancer cells) to treat cancer.

Ostomy Nurse – A nurse who specializes in the care of wounds or other openings in the skin. For bladder cancer patients who have their bladder removed, a stoma (see below) can be made in the skin to allow the passage of urine into a bag or through a catheter. An ostomy nurse can help survivors manage their stoma, provide advice about complications, and recommend the kinds of stoma care supplies that will work best.

Radiation Therapy – Treatment of cancer using radiation (energy that is directed to kill cancer cells) to control the growth of cancer or to relieve symptoms of cancer. Because of new discoveries, there are now many different kinds of radiation therapy. Some kinds are better for certain kinds of cancer than others. A radiation oncologist is a physician who can help decide whether radiation therapy is the right treatment and if so, what kind.

Recurrence – Cancer sometimes can come back after it's treated. When cancer comes back, it is referred to as a recurrence. Some types of cancer come back more frequently than others. Physicians use information like the stage to understand how likely a cancer is to come back.

Smoking Cessation – Bladder cancer is commonly related to being a smoker. After a person is diagnosed with bladder cancer, it is important that they stop smoking if they are still a smoker. Cancer centers and other hospitals can offer treatments to help smokers stop. These treatments usually include medication to reduce craving nicotine and counseling to help solve problems that may ruin efforts to quit.

Stoma – An opening in the skin where a person may pass urine after having their bladder removed. Urine may drain through the stoma into a bag that is attached to the person. Sometimes urine collects in a pouch inside the person's abdomen and the pouch is drained through the stoma with a catheter.

Survivorship – People affected by cancer need help with returning to their lives before their cancer diagnosis as much as they can. Health care providers and researchers who work in cancer survivorship work to reduce the impact of cancer on survivors using medical, counseling, and other treatments to improve the survivors' experience. Family, caregivers, and other loved ones are an important part of survivorship since they are also affected by cancer.

Systemic – Some conditions and treatments affect only certain parts of the body. Others are systemic, meaning they affect the person as a whole. Medical treatments are more likely to affect the whole person than surgical treatments.

Tumor Grade – A grade is assigned to determine the progress of cancer. Higher numbers are usually considered to be more advanced cancers.

Tumor Histology – Histology refers to the structure of the cancer cells. Understanding the structure can help physicians identify the best kind of treatments.

TURBT (Transurethral Resection of Bladder Tumor) – In early stage bladder cancer, the cancer cells can sometimes be removed by inserting a small instrument into the urethra (the opening where urine comes from) and scraping the cells from the side of the bladder. A TURBT is often combined with an intravesical therapy like BCG (see above).

Upper Tract – Refers to the upper portion of the urinary system which includes the kidneys and the ureters.

Ureter – A connection that carries urine from the kidneys to the bladder.

Urethra – The tube that carries urine from the bladder outside of the body. The urethral meatus is the opening from which urine comes. In males, semen also is passed through the urethra.

Urinary Diversion – A way of storing and removing urine from the body after the bladder is removed.

Urinary Tract – Refers to the kidneys, ureter, bladder and urethra. This system filters waste products from the body, and then creates, stores and evacuates urine.

Urinary Tract Infection (UTI) – An infection that affects one or more parts of the urinary tract.

Urologist – A physician trained to study and treat disease of the urinary and sexual health systems. Some urologists specialize in cancer and are referred to as urologic oncologists (see above). Others specialize in treating erectile dysfunction (see above) and other sexual health problems, incontinence (see above), or other diseases that affect urinary and sexual health.

Urostomy – See Stoma (above).
