# Supplementary material for: Development and evaluation of a bladder Cancer specific survivorship care plan by patients and clinical care providers: a multi-methods approach
Source: BMC Health Serv Res. 2020 Jul 24;20:686. doi: 10.1186/s12913-020-05533-7 (PMC7379822; doi:10.1186/s12913-020-05533-7)
Supplement: Supplementary file 3 — Additional file 3. Encounter Data Sheet. This file includes information pertaining to the logistics of completing the BC-SCP such as the provider type and the time taken to complete the BC-SCP, 8 item-survey with 7-point Likert response scale. [file 12913_2020_5533_MOESM3_ESM.pdf]

Bladder Cancer Survivorship Care Plan
Encounter Data Sheet

Completion Date	Completion **Start** Time	Completion **Stop** Time

Provider Name: ____

Provider Type	☐ Urologist ☐ Med Oncologist	☐ Nurse Practitioner/ Physician's Assistant/ Nurse – other	
	☐ Medical Assistant	☐ Resident/ Fellow	☐ Student/Volunteer
	☐ Social Worker	☐ Other: ____________________	
**Patient characteristics**	Gender	☐ Male	☐ Female
Age:	____________________		
Race/ethnicity	☐ White	☐ Black/African-American	☐ Asian
	☐ Hispanic/Latino	☐ Other	
Insurance Type	☐ Medicare	☐ Medicaid	☐ Private
	☐ VA	☐ Other	☐ None
Was the form completed in the presence of the patient?		☐ Yes	☐ No
Where was the care plan completed?	☐General patient room<br>☐Staff room	☐Procedure/treatment room<br>☐ Outside of clinic	☐Consultation room
Did care plan completion result in a higher billing code?		☐ Yes	☐ No
Number of new patient visits and other-visits seen in clinic today.		New Patients________ Other visits________	

Comments: Are there missing fields, revised language, or changes to the template organization that should be considered?Please also include general comments from providers or patients.

**Encounter Questions**	**Not at all**						**Very Much**
1. Was the information that was requested clear?	**1**	**2**	**3**	**4**	**5**	**6**	**7**
2. Was it difficult to locate the requested information?	**1**	**2**	**3**	**4**	**5**	**6**	**7**
3. Is this format (assuming further revision) one you would consider using in your practice?	**1**	**2**	**3**	**4**	**5**	**6**	**7**
4. Did the care plan completion hamper clinic flow? If yes, please comment above.	**1**	**2**	**3**	**4**	**5**	**6**	**7**
5. Do you currently have ample clinic resources to complete survivorship care plans in all new patients?	**1**	**2**	**3**	**4**	**5**	**6**	**7**
6. Was the patient an engaged and active participant in the completion of the care plan?	**1**	**2**	**3**	**4**	**5**	**6**	**7**
7. Did the care plan completion enhance the dialogue between you and the patient?	**1**	**2**	**3**	**4**	**5**	**6**	**7**
8. Did the patient appear interested in receiving the appendix portion of the care plan?	**1**	**2**	**3**	**4**	**5**	**6**	**7**
